# Comparing Emergent and Elective Colectomy Outcomes in Elderly Patients: A NSQIP Study

**DOI:** 10.1155/2021/9990434

**Published:** 2021-12-06

**Authors:** Mostapha El Edelbi, Ibrahim Abdallah, Rola F. Jaafar, Hani Tamim, Samer Deeba, Samer Doughan

**Affiliations:** ^1^Department of Surgery, American University of Beirut, Beirut, Lebanon; ^2^Clinical Research Institute, Biostatistics Unit, American University of Beirut, Beirut, Lebanon

## Abstract

**Introduction:**

With the increasing prevalence of colorectal cancer (CRC) worldwide, especially in the elderly, and the variability between physiological and chronological age and its impact on functional status, acute symptoms leading to emergent surgery due to colorectal malignancy may lead to increased morbidity and mortality. The aim of this study is to identify the outcome differences of elective vs. emergent open colectomy in patients above 80 years.

**Methods:**

The National Surgical Quality Improvement Program (NSQIP) database was reviewed from 2010 to 2014 for open colectomy based on CPT codes. Comparison between groups was done based on the clinical context at presentation as elective or emergent surgery. Data were analyzed using SAS.

**Results:**

Elective colectomies were performed in 8289 (70.8%) vs. emergent colectomies in 3409 (29.1%). Emergent colectomy patients had higher American Society of Anesthesiologists (ASA) preoperative classification III-IV, 1429 (42.0%) and 224 (6.6%), vs. 1238 (14.9%) and 21 (0.2%) in elective colectomy patients (*p* < 0.0001). Emergent colectomy patients had more comorbidities such as chronic obstructive pulmonary disorder (493 (14.5%) vs. 796 (9.6%)), congestive heart failure (206 (6.0%) vs. 310 (3.8%)), dialysis (106 (3.1%) vs. 56 (0.7%)), and acute renal failure (166 (4.9%) vs. 46 (0.6%)) (*p* < 0.0001), respectively. Postoperative morbidity and mortality were significantly higher in emergent colectomy (1651 (48.4%) and 872 (25.6%)) vs. elective colectomy (1859 (22.4%) and 567 (6.8%)) (*p* < 0.0001), respectively.

**Conclusion:**

Emergent open colectomy in elderly patients carries a higher risk of morbidity and mortality when compared to elective open colectomy with risk factors being higher ASA classification and more comorbidities.

## 1. Introduction

Colorectal cancer (CRC) is the third most common cancer and leading cause of cancer-related death in the United States in males and females [1]. Roughly 60% of CRC patients are above the age of 70 years at the time of diagnosis, and 43% are above the age of 75 years at diagnosis [2]. Aging can be defined as a natural occurring process leading to a decline in the functional health status and health reserve of multiple organ systems. There is profound variance in elderly patients when it comes to functional health status and frailty. An elderly patient's chronological age may not reflect the patient's physiological age, and vice versa. Physiological age, which most accurately represents a patient's health status, will depend on the patient's frailty, comorbidities, functional health status, and reserve. Colon cancer treatment modalities should take into consideration the patient's physiological age, functional health status, life expectancy, tolerance to treatment, and frailty since overtreatment may lead to the patient's demise [2]. Studies have shown that elderly patients who are not frail and have a good functional health status can be treated for CRC as aggressively as the younger population, while treatment in the elderly with increased frailty and a decrease in functional health status is still controversial [2]. Acute symptoms such as perforation, bleeding, and obstruction will convert an elective surgery into an emergent surgery. Acute symptoms that lead to an emergent surgery occur in up to 30% of patients with CRC [3–5]. Some studies have shown that advancing age is an independent risk factor and predictor for postoperative mortality [3–6]. Emergent colectomy in itself was associated with 20–30% increase in morbidity and mortality in elderly patients [7–19].

In addition to the context of surgery, other risk factors play an important role in increasing postoperative morbidity and mortality. Functional health status, frailty, comorbidities such as cardiorespiratory, metabolic, and renal diseases, and the American Association of Anesthesiologists (ASA) preoperative classification are predictors and independent risk factors for postoperative morbidity and mortality [7–9, 12, 16–19]. The goal of this study is to identify the outcomes of elective vs. emergent open colectomy in patients above the age of 80 years with the primary outcome being 30-day mortality and the secondary outcome being postoperative morbidity.

## 2. Methods

The American College of Surgeons National Surgical Quality Improvement Program (ACS-NSQIP) database was reviewed for open colectomy cases using Current Procedural Terminology (CPT) codes between the years 2010 and 2014. CPT codes are international standardized codes developed by the American Medical Association for medical and surgical procedures that are performed. The CPT codes that were used to search the NSQIP database and identify colorectal surgeries are detailed in Table 1. The inclusion criteria included all patients in the NSQIP database who were 80 years and older that underwent elective or emergent colorectal surgical intervention identified by the CPT codes 44140, 44146, 44150, 44155, and 44156. The exclusion criteria included all patients below the age of 80 who underwent colorectal surgical procedures not included in the specified CPT codes in Table 1. Patient demographics and characteristics were collected, and a comparison was made between the groups based on the patient's presentation labeled as elective or emergent. Colectomy performed on an elective basis was referred to as elective colectomy, and surgery performed in an emergency situation was referred to as emergent colectomy.

### 2.1. Variables

The following preoperative variables were included in the analysis: age, race, gender, history of diabetes mellitus, current smoking, hypertension, history of chronic obstructive pulmonary disease (COPD), history of congestive heart failure (CHF), body mass index (BMI) and weight loss, ASA classification, preoperative blood transfusion, preoperative total bilirubin, white blood count, hematocrit, platelet count, and international normalized ratio (INR) of prothrombin time (PT) values.

Operative variables including operative time were analyzed. Outcome variables included 30-day mortality, length of hospital stay, bleeding, respiratory, cardiac, and urinary complications, sepsis, thromboembolism, related readmission, and related 30-day reoperation.

### 2.2. Statistical Analysis

Patient characteristics were described and compared between elective and emergent surgery groups. Statistical analyses were done using Statistical Analysis Software version 9.4 (SAS 9.4). Categorical variables were reported as frequency and percentage, whereas continuous variables were reported by mean and standard deviation. Comparison between variables within the groups was done by chi-square and Fisher exact tests, and the mean of continuous variables among the two groups was compared using the independent *t*-test. Simple and multiple logistic regressions were used to analyze the association between variables and 30-day outcomes. Statistical significance was considered for two-sided *p* value <0.05.

## 3. Results

### 3.1. Patient Demographics and Characteristics

A total of 11698 patients who were 80 years or older that underwent open colectomy were identified in the ACS-NSQIP database. Patient preoperative demographic characteristics comparing elective versus emergent colectomies are summarized in Table 2. Elective colectomies were performed in 8289 (70.8%) patients, while emergent colectomies were performed in 3409 (29.1%) patients. The mean age of the patients who underwent elective colectomy was 84.5 ± 3.5 years while in patients who underwent emergent colectomy was 84.9 ± 3.3 years (*p*=0.3). Patients who underwent emergent colectomy had a significantly higher ASA classification III-IV, with 1429 (42.0%) and 224 (6.6%) being classified as ASA III/IV, in comparison to 1238 (14.9%) and 21 (0.2%) for patients who underwent elective colectomy. Patients who underwent elective colectomy had a relatively better functional health status (*p* < 0.0001) as compared to patients who underwent emergent colectomy.

There was no difference between the two groups based on gender; however, a significant difference was observed based on BMI, smoking history, and more than 10% weight loss in the last 6 months (*p*=0.005, *p*=0.02, and *p* < 0.001, respectively). Data about patient demographics and comorbidities are presented in Table 2.

A significant difference in comorbidities was observed in patients who underwent emergent colectomy as compared to elective colectomy such as COPD (493 (14.5%) vs. 796 (9.6%)), history of CHF (206 (6.0%) vs. 310 (3.8%)), patients on dialysis (106 (3.1%) vs. 56 (0.7%)), acute renal failure (166 (4.9%) vs. 46 (0.6%)), and hypertension requiring medication (2609 (76.5%) vs. 6182 (74.6%)) (respectively, all *p* < 0.0001 except hypertension, *p*=0.03).

Steroid use for chronic conditions, bleeding disorders, blood transfusions before surgery, and sepsis (1673 (49.2%) vs. 604 (7.3%)) within 48 hours of surgery were significantly more prominent in patients undergoing emergent colectomy in comparison to patients who underwent elective colectomy (*p* < 0.0001).

Diabetes mellitus, dyspnea at rest or on moderate exertion, and disseminated cancer were found to be higher in patients who underwent elective colectomy as compared to emergent colectomy (*p*=0.02,  *p*=0.04,  and *p* < 0.0001, respectively).

Preoperative laboratory values were worse except for hematocrit for patients who underwent emergent colectomy. For emergent colectomy, preoperative bilirubin was higher (0.8 ± 0.7 vs. 0.6 ± 0.6), white blood count (WBC) was higher (13.1 ± 9.3 vs. 8.2 ± 3.9), platelet count was lower (245.9 ± 110.6 vs. 260.9 ± 99.7), and preoperative INR was higher (1.4 ± 0.7 vs. 1.2 ± 0.3) in the patients who underwent elective colectomy (*p* < 0.0001).

### 3.2. Postoperative Outcomes

Postoperative outcome measures of elective colectomies versus emergent colectomies are shown in Table 3. Mortality was significantly higher in the patients who underwent emergent colectomy (872 (25.6%)) when compared to patients who underwent an elective colectomy (567 (6.8%)) (*p* < 0.0001). Return to the operating room was higher for patients who underwent emergent colectomy (928 (9.9%)) as compared to patients who underwent an elective colectomy (492 (5.9%)) (*p* < 0.0001). Bleeding requiring transfusion was also significantly higher in the patients who underwent emergent colectomy (928 (27.2%)) compared to the patients who underwent elective colectomy (1476 (17.8%)) (*p* < 0.0001). Readmission related to the initial surgical procedure was higher for patients who underwent elective colectomy (583 (10.2%)) compared with patients who underwent emergent colectomy (205 (8.4%)) (*p*=0.009).

Postoperative morbidity was significantly higher in the patients who underwent emergent colectomy with 1651 (48.4%) patients experiencing postoperative morbidity as compared to patients who underwent an elective colectomy (1859 (22.4%)) (*p* < 0.0001). Of the postoperative morbidities, 894 (34.1%) (*p* < 0.0001) patients who underwent emergent colectomy suffered from postoperative sepsis, and 1010 (29.6%) (*p* < 0.0001) experienced postoperative respiratory morbidity, while patients who underwent elective colectomy experienced sepsis (693 (8.4%)) and respiratory morbidity (857 (10.3%)). Postoperative cardiac, urinary, and wound-related morbidities and thromboembolism were higher in patients who underwent emergent colectomy. Cardiac morbidity in patients who underwent emergent colectomy was 243 (7.1%) (*p* < 0.0001), urinary morbidity was 157(4.9%), and wound-related morbidity was 300(8.8%) (*p* < 0.0001), and thromboembolism was 150 (4.4%) (*p* < 0.02). As for patients who underwent elective colectomy, postoperative cardiac morbidity was 264 (3.2%), urinary morbidity was 169 (2.0%), wound morbidity was 550 (6.6%), and thromboembolism was 288 (3.5%).

Multivariate analysis of 30-day postoperative outcome measures of elective colectomies versus emergent colectomies is presented in Table 4. Patients undergoing emergent colectomy had a significantly increased mortality rate that was 4.76 times higher (95% CI: 4.21–5.39) and an increased composite morbidity rate that was 3.12 times higher (95% CI: 2.85–3.42) than patients who underwent elective colectomy (*p* < 0.0001). Emergent colectomy patients were 1.27 (95% CI: 1.02–1.58) (*p*=0.03) times and 1.33 (95% CI: 1.13–1.55) (*p*=0.0005) times more likely to develop postoperative thromboembolism and postoperative wound complications as compared to elective colectomy patients, while postoperative sepsis (odds ratio (OR): 3.59; 95% CI: 3.19–4.04), respiratory (OR: 3.68; 95% CI: 3.30–4.10), cardiac (OR: 2.44; 95% CI: 2.02–2.96), and urinary (OR: 2.44; 95% CI: 1.93–3.07) complications were significantly more likely to occur in patients who underwent emergent colectomy (*p* < 0.0001).

Length of hospital stay was significantly longer for emergency colectomy patients (14.71 ± 13.29 days) when compared to elective colectomy patients (11.74 ± 9.27 days) (OR: 2.97; 95% CI: 2.51–3.43) (*p* < 0.0001). Patients who underwent emergent colectomy had 1.77 (95% CI: 1.60–1.95) times higher incidence of bleeding and were 1.82 (95% CI: 1.56–2.13) times more likely to return to the operating room when compared to patients who underwent elective colectomy (*p* < 0.0001). Readmission related to the initial surgery in patients who underwent elective colectomy was 19% (OR: 0.81; 95% CI: 0.67–0.97) less than patients who underwent emergent colectomy (*p*=0.02).

## 4. Discussion

With the baby boomers born between 1946 and 1964 in the United States of America currently aged 55 to 73 years in 2019, this aging population will have an increased incidence of colorectal malignancy which presents a unique challenge to the healthcare system. The number of Americans above the age of 65 is projected to double from 52 million in 2018 to 95 million in 2060 and a rise in the population share from 16% to 23% [20]. As life expectancy increases over time due to improvement in healthcare, this will lead to an aging population where the incidence of CRC will increase with age in the elderly. With CRC being the third most common cancer in the United States of America among males and females as well as the third leading cause of cancer-related death in the United States in both men and women [1], it represents a disease with an increased morbidity and mortality in the elderly population especially when treated in an emergency setting.

Emergent colectomy performed in the elderly patient above the age of 80 can be problematic. Such patients already have certain risk factors that pose an increased risk for postoperative morbidity and mortality. Elderly patients often have associated comorbidities, are increasingly frail, and have a decreased functional health status that predisposes them for postoperative complications, which will not be tolerated well by the patients due to their limited physiologic reserve. Emergent surgery by its very nature is associated with increased morbidity and mortality [7, 9–19]. More so, when such patients with multiple preoperative risk factors are taken to surgery in an urgent manner due to acute symptoms, where there is an inadequate amount of time to preoperatively optimize these patients, this will increase the risk for postoperative morbidity and mortality. The decision to proceed with an emergent surgery in the elderly should account for multiple factors such as the risks and benefits of the surgery, the expected postoperative quality of life, intensive unit stay, and the patient's wishes and desires.

Functional health status, comorbidities such as cardiorespiratory, metabolic, and renal diseases, and ASA classification are predictors and independent risk factors for postoperative morbidity and mortality [7–9, 12, 16–19]. In our study, patients with a decrease in their functional health status (25.8%) (*p* < 0.0001) made up a larger portion of the patients who underwent emergent colectomy when compared to the same group in the elective colectomy (*p* < 0.0001). Our study also showed that patients who were undergoing emergent colectomy ultimately had an increased postoperative morbidity (*p* < 0.0001) and mortality (*p* < 0.0001) as well as significantly more preoperative cardiopulmonary, renal, and hematologic comorbidities as compared to the elective colectomy patients. Our data also showed that postoperative morbidity was higher in the emergent colectomy patients with substantial postoperative morbidity due to cardiac (7.1%) (*p* < 0.0001), pulmonary (29.6%) (*p* < 0.0001), and renal (4.9%) (*p* < 0.0001) diseases. Turrentine et al. demonstrated that emergent operations in patients older than 80 years increased morbidity from renal, cardiac, and respiratory occurrences [21].

Frailty is defined as a decrease in functional reserve due to physiologic multisystem deterioration due to age [22]. A study conducted by Neuman et al. demonstrated that overall survival in patients above 80 years of age after elective colectomy for colon cancer for frail vs. nonfrail patients was 58.8% versus 95.0% at 90 days and 41.5% versus 87.7% at 1 year and that frailty had the strongest association with mortality [9]. Obeid et al. demonstrated that frailty index remained an independent predictor of Clavien class IV and V complications as did open colectomy and ASA score 4 [16]. Congiusta et al. were able to demonstrate that increasing frailty caused a statistically significant increase in rates for 30-day mortality, Clavien–Dindo grade >3, reintubation, ventilator >48 hours, and reoperation in patients undergoing open emergent colectomy [18].

The data from our study showed that postoperative mortality in emergent colectomy patients was significantly higher (25.6% vs. 6.8%) (*p* < 0.0001) when compared to the patients undergoing elective colectomy. Postoperative composite morbidity was also significantly higher in the emergent colectomy patients (48.4% vs. 22.4%) (*p* < 0.0001) when compared to patients who underwent elective colectomy. Morse et al. demonstrated similar outcomes when comparing elective (*n* = 65) and emergent (*n* = 39) colectomies with the results showing significant differences in morbidity (20% vs. 51%, *p* < 0.001) and 30-day mortality rates (7.7% vs. 30.7%, *p* < 0.005) [10]. A British study conducted by Kurian et al. with 162 patients above 80 years of age, of which 99 patients underwent emergent colectomy, showed that postoperative acute renal failure (3% vs. 19%, *p*=0.0032) and in-hospital deaths were significantly higher (4.7% vs. 28%, *p*=0.0002) among the patients undergoing emergent colectomies and that 6-month mortality significantly increased to 52% in the aforementioned patients [13]. A similar British study conducted by Mamidanna et al. grouped patients into age groups with group C above 80 years of age who underwent emergent colectomies, where the study showed that cardiac and respiratory complications were significantly higher in group C with 1-year mortality reaching 51.2% [14].

Surgical treatment and clinical outcomes in elderly patients above 80 with CRC remain controversial. Faiza et al. showed that advancing age itself was an independent predictor of postoperative mortality in elderly patients above 75 undergoing colectomies for colon cancer [6]. Kolfschoten et al. showed that, in elderly patients over the age of 80, the mortality rate increased with age for both elective and emergent colectomies [7]. The same study showed that mortality was significantly increased when risk factors such as ASA III, procedure, and age were independent predictors of mortality in patients above 80 years [7]. With all the literature studies supporting the fact that emergent colectomy in elderly patients above the age of 80 has an increased risk of morbidity and mortality, some authors have suggested the possibility to delay surgery if the source of the acute symptoms can be controlled. The most common presenting acute symptom of CRC is obstruction. Decompression with a colonic stent followed by an interval elective colectomy may be safe and may show a reduction in postoperative morbidity and mortality [10–12, 23–25].

The limitations of our study are that it is a retrospective cohort study based on a large predetermined dataset where certain data and variables are not included or available. Another limitation is differential loss to follow-up which may increase bias. Potential confounding factors can be missed due to the retrospective nature of the data.

In conclusion, postoperative morbidity and mortality are significantly higher in elderly patients above the age of 80 who underwent emergent colectomy as compared to the same population who underwent elective surgery. Across the literature, a significant predictor of postoperative morbidity and mortality in elderly patients undergoing colectomy was ASA classification III and IV [7–9, 12, 16–19]. Elderly patients who present with acute symptoms requiring emergent colectomies are likely to be more comorbid, with a higher ASA classification, increasing frailty, and a declining functional health status which are all predictors for increased morbidity and mortality as our study has shown. Temporizing factors that may delay surgery in order to optimize patients as well as transform an emergent surgery into a more controlled elective surgery may be the optimal choice for elderly patients. The choice of surgery should be based on multiple factors that need to be discussed clearly with the patient and the patient's family. Such factors are the increased risk of morbidity and mortality due to emergent colectomy in the elderly patient, postoperative intensive unit stay, postoperative quality of life, and the patients' desires and wishes. This study demonstrates clear risk factors for postoperative morbidity and mortality in patients above the age of 80 presenting for emergent colectomy as compared to elective cases, which can help in decision-making and discussions with elderly patients presenting with acute symptoms.

## Figures and Tables

**Table 1:** Current Procedural Terminology codes with the associated surgical procedure.

CPT code	Surgical procedure
44140	Colectomy partial with anastomosis
44146	Colectomy partial with coloproctostomy (low pelvic anastomosis) with colostomy
44150	Colectomy, total, abdominal, without proctectomy, with ileostomy or ileoproctostomy
44155	Colectomy, total, abdominal with proctectomy, with ileostomy
44156	Colectomy, total, abdominal, with proctectomy, with continent ileostomy

**Table 2:** Preoperative baseline patient characteristics' comparison of elective colectomies versus emergent colectomies.

	Elective	Emergent	*p* value
	*n* = 8289	*n* = 3409	
Gender			
Male	3285 (39.67%)	1300 (38.13%)	0.1339
Female	4996 (60.33%)	2105 (61.82%)	
Age	84.55 ± 3.31	84.95 ± 3.36	
Diabetes mellitus	1550 (18.70%)	577 (16.93%)	
BMI	26.08 ± 5.45	25.47 ± 5.68	
Current smoker within one year	394 (4.75%)	197 (5.78%)	0.0214
Functional health status			
Independent	7235 (87.57%)	2504 (74.24%)	<0.0001
Partially dependent	896 (10.84%)	588 (17.43%)	
Totally dependent	131 (1.59%)	281 (8.33%)	
History of COPD	796 (9.60%)	493 (14.46%)	<0.0001
History of CHF	310 (3.75%)	206 (6.04%)	<0.0001
Dialysis	56 (0.68%)	106 (3.11%)	<0.0001
Acute renal failure	46 (0.55%)	166 (4.87%)	<0.0001
Disseminated cancer	585 (7.06%)	172 (5.05%)	<0.0001
Dyspnea at rest or on moderate exertion	1242 (14.98%)	461 (13.52%)	0.0418
Hypertension requiring medication	6182 (74.58%)	2609 (76.53%)	0.0264
>10% weight loss in the last 6 months	588 (7.09%)	182 (5.34%)	0.0005
Blood transfusion before surgery	566 (6.83%)	401 (11.76%)	<0.0001
ASA classification			
I	1456 (17.59%)	243 (7.15%)	<0.0001
II	5564 (67.21%)	1504 (44.24%)	
III	1238 (14.95%)	1429 (42.03%)	
IV	21 (0.25%)	224 (6.59%)	
Steroid use for chronic conditions	349 (4.21%)	231 (6.78%)	<0.0001
Bleeding disorders	717 (8.65%)	686 (20.12%)	<0.0001
Transfusion requirement 72 hours prior to surgery	566 (6.83%)	401 (11.76%)	<0.0001
Sepsis within 48 hours of surgery	604 (7.31%)	1673 (49.15%)	<0.0001
Preop total bilirubin	0.62 ± 0.64	0.79 ± 0.72	<0.0001
Preop WBC	8.16 ± 3.88	13.15 ± 9.29	<0.0001
Preop hematocrit	34.22 ± 5.33	35.10 ± 6.70	<0.0001
Preop platelet count	260.93 ± 99.66	245.93 ± 110.59	<0.0001
Preop INR	1.14 ± 0.29	1.37 ± 0.67	<0.0001

**Table 3:** Univariate analysis: 30-day postoperative outcome measures of elective colectomies versus emergent colectomies.

	Elective	Emergent	*p* value
	*n* = 8289	*n* = 3409	
Mortality	567 (6.84%)	872 (25.58%)	<0.0001
Number of bleeding transfusion occurrences	1476 (17.81%)	928 (27.22%)	<0.0001
Return to the operating room	492 (5.94%)	339 (9.94%)	<0.0001
Readmission, related	583 (10.24%)	205 (8.39%)	0.0096
Composite morbidity	1859 (22.43%)	1651 (48.43%)	<0.0001
Thromboembolism	288 (3.47%)	150 (4.40%)	0.0166
Wound	550 (6.64%)	300 (8.80%)	<0.0001
Cardiac	264 (3.18%)	243 (7.13%)	<0.0001
Respiratory	857 (10.34%)	1010 (29.63%)	<0.0001
Urinary	169 (2.04%)	167 (4.90%)	<0.0001
Sepsis	693 (8.36%)	894 (34.09%)	<0.0001

**Table 4:** Multivariate analysis: 30-day postoperative outcome measures of elective colectomies versus emergent colectomies.

	Elective	Emergent	OR ^*∗*^ (95% CI ^*∗*^ ^*∗*^)	*p* value

Mortality	490 (6.9)	758 (26.0)	4.76 (4.21–5.39)	<0.0001
Bleeding	1265 (17.7)	804 (27.6)	1.77 (1.60–1.95)	<0.0001
Return to OR	428 (6.0)	304 (10.4)	1.82 (1.56–2.13)	<0.0001
Readmission, related	471 (10.4)	167 (8.6)	0.81 (0.67–0.97)	0.02
Composite morbidity	1630 (22.9)	1401 (48.1)	3.12 (2.85–3.42)	<0.0001
Thromboembolism	249 (3.5)	128 (4.4)	1.27 (1.02–1.58)	0.03
Wound	479 (6.7)	254 (8.7)	1.33 (1.13–1.55)	0.0005
Cardiac	225 (3.2)	215 (7.4)	2.44 (2.02–2.96)	<0.0001
Respiratory	749 (10.5)	879 (30.2)	3.68 (3.30–4.10)	<0.0001
Urinary	151 (2.1)	146 (5.0)	2.44 (1.93–3.07)	<0.0001
Sepsis	603 (8.5)	726 (24.9)	3.59 (3.19–4.04)	<0.0001
LOS	11.74 ± 9.27	14.71 ± 13.29	2.97 (2.51–3.43)	<0.0001

^*∗*^Odds ratio. ^*∗∗*^Confidence interval.

## Data Availability

All the information in the article is part of the American College of Surgeons National Surgical Quality Improvement Program (ACS-NSQIP) database. Access can be granted following the steps mentioned on their website (https://www.facs.org/quality-programs/acs-nsqip).
